# Syphilization

**Published:** 1857-06

**Authors:** 


*Syphilization.*—The medical reader will remember that a few years since the startling announcement was made of a discovery in France, of a means of controlling the ravages of syphilis by a process of inoculation—the disease itself was not only to be treated and cured by this method when present, but the system was to be rendered invulnerable to all subsequent attacks by the same operation, in the same. manner as vaccination protects the system against the contagion of small-pox.

The proposition was a bold one, and while it challenged attention, the utmost faith in the probable triumphs of science and skill over the domains of disease, could scarce give credence to the claims of this discovery. The mind revolted at the proposition,—there was the danger, and the probability of permanently contaminating the system with one of the most horrid diseases with which humanity is cursed; and every sentiment of morality was shocked as it saw in this pretended discovery a full license given to an indulgence in the grossest licentiousness, unchecked by a single fear which ordinary caution now imposes. In France itself the subject met strong opponents, and the authority of Ricord's name was brought to bear in opposition to it. It required bold men to urge it, and in the light of the melancholy results of some unsuccessful attempts to verify the truth of the claims put forth, the whole subject seemed very speedily to pass from notice and attention, at least so far as this country, England and France were concerned. But such seems not to have been its fate elsewhere. Upon the continent of Europe, in various places, the subject has attracted the attention of earnest minds bold enough to pursue the inquiry, and has been put to the test of experience, and the results are gradually being disclosed to the world. These experiments have, from the moment of the first announcement of the discovery, been perseveringly prosecuted in Norway, in Sweeden, in Turin and elsewhere. Professor Boeck in Christiana, Danielson in Bergen, Carlsson in Stockholm, and Sperino in Turin, have for some years, been engaged in a series of careful experiments and observations made to test the truth of the claims set up by the originator of this practice.

The attention of the English reader will be renewedly called to this matter, by a notice in the April number of the *British and Foreign Medico-Chirurgical Review* of the present year, of two volumes recently published by Prof. Wm. Boeck, Professor of Medicine in the University of Norway, detailing the results of his experiments in syphilization up to the summer of 1856. These experiments have been carried on in a large hospital, in the presence of intelligent colleagues, and of a large body of students; and with so many witnesses to the truthfulness of the statements but little room is left for doubts as to their correctness. The character of the author seems, moreover, to be above suspicion.

The article in the *Review* contains a historical sketch of the origin and progress of syphilization, and we deem the subject one of sufficient importance to warrant us in giving an epitome of the said article.

We know of no other source from whence to derive the information there given, and from present appearances the profession will be compelled to meet this question despite of any considerations of morality which may surround it.

Auzias Turenne, a young French physician, was the originator of this novel practice. As early as the year 1844, it seems that Turenne commenced a series of experiments in order to test the truth of the doctrines of John Hunter, of the non-communicability of syphilis to the lower animals. After many experiments and several failures, he succeeded in producing in monkeys inoculated with chancre matter, a disease which had all the characteristics of true chancre. This was transferred to rabbits, cats and horses, and from them was returned by inoculation to the human subject, the first trials in this regard being made by Dr. Robert Weltz, of Würzburg, on his own person.

While thus engaged in researches on the transmission of syphilis to animals, Turenne became aware of the curious fact, that each succeeding chancre produced by inoculation, became less and less in each animal, until at length a period arrived when inoculation apparently lost its power, and no chancres, or sores of any kind, followed the application of the venereal virus. From these facts he drew the inference, that by prolonged inoculation with the syphilitic poison, a constitutional state, or diathesis, was at length produced, in which the system was no longer capable of being affected by syphilis. This condition he terms "syphilization," and upon this asserted discovery all the subsequent experiments and peculiar mode of treatment are based.

Turenne's doctrines were laid before the French Academy of Medicine, November 18, 1850, and as was to be expected, met with the most vehement opposition. He had only recently commenced his experiments on syphilization in the human subject, and he had, therefore, few or no data for the support of his opinions, and he not only proposed to employ syphilization for the cure of primary and secondary syphilis, but suggested its use as a prophylactic against the contagion of the disease in persons uncontaminated by the poison. The discussion mainly turned upon the latter proposition, nd the indignation of his opponents was unbounded at the audacity and immorality of such a proposal. A few were found to urge a careful investigation of the subject, but the weight of the opposition was too great for them to contend against, and the proposals of Turenne were unequivocally condemned. In England the subject excited but little interest, being casually noticed in some few of the medical journals.

While the subject was thus disposed of in Paris, the question was made one of careful study by Sperino, of the city of Turin, a physician attached to the Veneral Hospital of that place. He made his first report on the subject to the Medico-Chirurgical Academy of Turin, on the 23d of May, 1851. In this report he gives the full details of fifty-two cases thus treated by him at the hospital. The subjects were all prostitutes, and all suffering from aggravated forms of primary or secondary syphilis. The results were, that after frequent and· repeated inoculation of the virus, the contagious matter ceased to produce any effect whatever, and contemporaneously with the establishment of this epoch all former ulcers had healed, and buboes, enlargement of bones, cutaneous stains or blotches, had either disappeared altogether, or were rapidly going away.

Sperino's observations were confirmed by similar results obtained by Dr. Gamberini, at Bologna, and by Gulligo, at Florence. In 1853, Sperino published a detailed account of 96 cases of syphilization, in a bulky volume of 903 pages.

Of these 96 cases, 53 were of primary syphilis, and 43 of constitutional disease. Fifty of the primary cases were cured, 2 failed, and 1 was not treated by syphilization alone.

Of the 43 cases of constitutional disease, 26 were treated by syphilization alone, and 17 by this method in conjunction with mercury or iodine.

Of the 26 cases treated by syphilization, 25 were said to have been cured.

In only 2 cases of primary disease did any constitutional symptoms appear, and those symptoms rapidly yielded under a continuance of the syphilization. No relapse has yet taken place in any case, and many of these were of very severe character, and were not likely to heal spontaneously.

In confirmation of all this testimony to the validity of Turenne's claims, we have the published reports of Dr. Boeck, of the University of Norway. The same plan of treatment is now pursued by Dr. Danielson, in the hospital at Bergen, and he endorses the results obtained by Dr. Boeck.

Dr. Boeck holds, that as a prophylactic syphilization is *unjustifiable*, and says that even its discoverer now holds this opinion. Syphilization, he says, can therefore only be adopted where venereal disease already exists; but even here, in his opinion, it is far from being applicable to every case. He believes it to be contra-indicated in the primary form. In ordinary non-indurated chancres, he would not practice syphilization, for there is a strong probability that in such cases, the patient will escape the constitutional affection altogether. He does not, however, subscribe implicitly to Ricord's opinion, that a non-indurated chancre can never give rise to constitutional symptoms. He seems disposed to wait until constitutional symptoms declare themselves before he subjects his patients to a course of treatment which is necessarily long and painful.

The conclusions drawn by Dr. Boeck, from the eighty-four cases of syphilization which he had treated up to March, 1856, are as follows:

I. That in all cases, without exception, immunity to the venereal virus is obtained, sooner or later, by inoculation of this poison.

II. That the symptoms of syphilis present at the commencement of syphilization, disappear during the employment of this mode of treatment.

III. That the general health does not suffer in the least from syphilization — on the contrary, if the patient has been in weak health before inoculation, he most materially improves in strength and appearance during the process.

Dr. Boeck says, that the time required to produce immunity depends not only on the variable strength of the virus — upon the rapidity or otherwise with which the inoculations succeed each other, and upon the number of chanchres — but also upon the idiosyncrasy of the individual. In one case immunity was obtained only after seventy-one chancres, but he regards the virus employed on this occasion as remarkably weak. He has been led to believe that great variety exists in the strength of the venereal virus, and his observations go to prove that the virulence of the poison varies in different countries. He thinks that the virulence of syphilis is rapidly diminishing in Norway. He has found the greatest difficulty in obtaining fresh chancre matter of sufficient power; and he has been compelled to depend upon matter taken from chancres incurred in Germany or in England.

In reply to the question, how long the immunity to the virus thus obtained last, whether like the result of vaccination for small-pox, it lasts for life, he is unable to answer. He inclines to the belief that the protection is effectual and permanent. He seems extremely cautious, and has not felt justified in putting those healed by syphilization to the proof of inoculation, for fear the treatment had returned the system back to its original condition of health, and freedom from contamination, and that an inoculation *after* the cure, *might again* produce constitutional symptoms.

The previous use of mercury, seems to have complicated the results of the treatment by syphilization. In some cases the cure was impossible without the use of iodine, which he regards as essentially an anti-mercurial. He regards the patient when treated by syphilization alone, as much less liable to relapses than when treated by mercurials.

Various methods of inoculating the venereal virus have been adopted by the advocates of this system. Turenne at first kept up a succession of single chancres; while Sperino made three or four separate inoculations at once, and repeated these two or three times in the week. After having in this way reached the number of twenty-four or thirty inoculations in all, he found that the chancres last produced were exceedingly small, and he then diminished the intervals, and made more inoculations at each siting. He found that the first chancres were deeper, larger, and more inflamed than those which succeeded them; and that by diminishing the intervals and increasing the number of inoculations, the earliest chancres visibly diminished, and were less painful and inflamed. To test this still further, Sperino ventured upon as many as sixty inoculations at once upon the same individual; but the result obtained was, that *immunity* to further inoculation set in before the syphilitic symptoms were cured, and relapses of the disease frequently ensued. He, therefore, returned to his former plan, and now inoculates for six or ten chancres at each sitting. While these chancres are progressing, it is neither necessary nor advisable to inoculate afresh, nor should this be done until the former chancres be developed. Should the chancres be developed two freely, and threaten to produce active inflammation, or to extend as phagedænic sores, he checks their progress by inoculating afresh at shorter intervals.

The practice of Dr. Boeck differs very little from that of Sperino. At first, afraid of producing too serious an impression upon the system, Dr. Boeck inoculated for two chancres only every six days, selecting that period of time because he found from experience that it required about five days to produce induration in a chancre; he does not, however, regard this latter circumstance absolutely essential. Subsequently he has shortened his intervals to three days, and increased the number of inoculations to eight or ten. Sperino places his punctures on the lower part of the abdomen, while Dr. Boeck prefers inoculating the arms and thighs.

We have given as fully as our limits will permit, the pith of the article contained in the *Review*. At the risk of a charge of plagiarism we have employed its language when convenient. The reports of cases given, and the arguments used to enforce the truth of assumed positions we are compelled to omit.

It would seem that this subject is now fully before the profession, and there is no evading the questions presented in its consideration. With the physician the subject is one of therapeutics and not of morals. The claims of the discover are true or false. This point can be established by experiment only. If true, the value of the discovery as a safe and desirable treatment, is next to be established by experience, and by that alone. It would seem at least a matter of prudence to withhold any further denunciations, and to cease all efforts to cover the subject under the weight of great names arrayed in opposition.

If further experience establishes all that is claimed for syphilization, the discovery is one of the most remarkable in the history of medicine, and it will assume a place next in rank with the unexplained mysteries of vaccination. J. M. N.

